# Complex magnetic structure and magnetocapacitance response in a non-oxide NiF_2_ system

**DOI:** 10.1038/s41598-019-39083-8

**Published:** 2019-03-01

**Authors:** S. Arumugam, P. Sivaprakash, Ambesh Dixit, Rajneesh Chaurasiya, L. Govindaraj, M. Sathiskumar, Souvik Chatterjee, R. Suryanarayanan

**Affiliations:** 1 Center for High Pressure Research, School of Physics, Bharathidasan University, Tiruchirappalli, 620 024 India; 2 Department of Physics & C for Solar Energy, Indian Institute of Technology Jodhpur, 342 037 Jodhpur, India; 3 UGC-DAE Consortium for Scientific Research, Kolkata Centre, Kolkata, 700 098 India; 4 Retired ICMMO, University of Paris- Sud, Orsay, 91405 France; 5 Present Address: 3, allée des Marronniers, Les Ulis, 91940 France

## Abstract

We report here on the complex magnetic structure and magnetocapacitance in NiF_2_, a non-oxide multifunctional system. It undergoes an anti-ferromagnetic transition near 68.5 K, superimposed with canted Ni spin driven weak ferromagnetic ordering, followed by a metastable ferromagnetic phase at or below 10 K. Our density functional calculations account for the complex magnetic structure of NiF_2_ deduced from the temperature and the field dependent measurements. Near room temperature, NiF_2_ exhibits a relatively large dielectric response reaching >10^3^ with a low dielectric loss of <0.5 at frequencies >20 Hz. This is attributed to the intrinsic grain contribution in contrast to the grain boundary contribution in most of the known dielectric materials. The response time is 10 μs or more at 280 K. The activation energy for such temperature dependent relaxation is ~500 meV and is the main source for grain contribution. Further, a large negative magneto capacitance >90% is noticed in 1 T magnetic field. We propose that our findings provide a new non-oxide multifunctional NiF_2_, useful for dielectric applications.

## Introduction

The dielectric permittivity (DP), materials have attracted broad attention for the realization of modern electronic devices with miniaturization, integration, high performance offering potential applications for smaller and faster electronics as well as high energy density storage^1–4^. Usually, high dielectric constant and low loss are required for those applications. A large effort is being devoted to the development and characterization of dielectric materials. Ideally, a DP material should exhibit a high dielectric constant in conjunction with the very low dielectric loss. In the present scenario, it is a challenging task to discover such materials destined for the applications of energy storage at room temperature. Recently, there have been several interesting reports on many excellent functional materials, such as doped BaTiO_3_, CaCu_3_Ti_4_O_12_ (CCTO), doped NiO, Bi_0.5_Na_0.5_TiO_3_, Ni_0.5_Zn_0.5_Fe_2_O_4_ and ZnO etc^1^. The observation of functional properties such as high dielectric constant, magnetocapacitance and magnetodielectric properties have been attributed to the existence of magnetic or non-magnetic semiconducting grains and insulating grain boundaries in these materials. The relaxation in these materials is attributed to the various factors and among these Maxwell – Wagner type relaxation process is observed in multigrain systems^5,6^. So far several oxide materials have been proposed as the possible multifunctional candidates. For example, the compound CaCuTi_3_O_12_ (CCTO) has a very high value of dilectric constant (~10^5^) with a low dielectric loss (>0.2). In fact, Subramanian *et al*. reported that CCTO showed the highest dielectric constant among all the investigated compounds of ACu_3_Ti_4_O_12_ (A = La, Ce, Pr, Nd, Sm, Eu, Gd, Tb, Dy, Ho, Er and Tm). Shanming Ke *et al*. observed a high value of 10^4^ in TiO_2_ co-doped with In and Nb at low dielectric loss (<0.05) values. It exhibits temperature and frequency independence over a broad range^7–10^. Recently, GaAs^11^ is identified as a new type of non-oxide dielectric material with a dielectric constant value around 10^3^. Transition metal difluorides (MF_2_ (M = Ni, Fe, Mn, Co)) are an important family of complex non-oxide functional materials, which garnered much interest earlier to understand the magnetic structure^12^. The magnetic properties of transition metal difluorides depend on the transition metal cations and lead to complex magnetic structure and spin coupled other ferroic properties^13^. The complex magnetic structure may provide an opportunity to investigate the interplay of the unpaired d-electrons for manipulating the magnetic properties in these materials. In conjunction with these studies, transition metal difluorides are studied as the cathode materials for rechargeable lithium batteries, preferably due to the intrinsic high ionic conductivity and possibility to adopt lithium at high atomic fractions, important for high power densities. In spite of such interesting materials properties and potential for energy storage, very little has been investigated to understand the functional properties in these materials^14,15^. These materials exhibit a rutile structure with P4_2_/mnm space group. The crystal structure of NiF_2_ has two molecules and six atoms per unit cell^16^. The anisotropy of the paramagnetic (PM) susceptibility showed below the Neel temperature (T_N_) of NiF_2_, the spins align perpendicular to the c-axis and the net magnetic moment along *a* and *b* axis. This is attributed to the rutile type crystal structure with two kinds of cation sites: one at corner and the other at the body center site in the unit cell^17^. Also, it develops a small orthorhombic distortion below the T_N_ point (=68.5 K) and shows week ferromagnetic moment below its transition point. The Ni atoms occupy high symmetry 2a Wyckoff positions and F atoms occupy 4f Wyckoff positions. The arrangement of F^−^ ions surrounding Ni^2+^ site forms a distorted octahedral in terms of four long and two short Ni–F bond lengths, leading to deviations in F-Ni-F bond angles from 90° ^18^. The nature of weak ferromagnetism (FM) is attributed to either transverse or longitudinal week FM in anti-ferromagnetic systems. Because the arrangements of magnetic moments correspond to the fact that when antiferromagnetic (AFM) vector L is rotated in the (110) plane towards the direction of the applied magnetic field^19,20^.

This study explores the detailed AFM phase at or below transition point T_N_ = 68.5 K. Further, we observed that a weak FM is superimposed in this AFM phase, followed by a metastable FM transition at ~10 K. The temperature and frequency dependent dielectric properties are investigated to understand the dielectric properties of the system. For the first time, we report here on a non–oxide multifunctional transition metal difluoride NiF_2_ exhibiting a dielectric permittivity of 10^3^ as well as a low dielectric loss (≤0.5) in the low-frequency range (20 Hz) at room temperature. Further we have observed a large negative magneto capacitance >90% under a magnetic field of 1 T even at room temperature. We propose that a new non-oxide multifunctional NiF_2_, may be useful for magnetic, dielectric, and magnetocapacitance applications.

## Results

**Figure 1:**
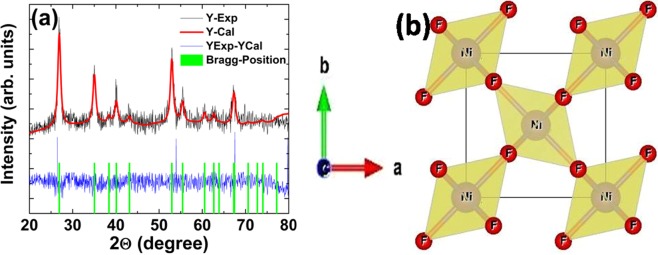
(**a**) The diffraction diagrams of NiF_2_ at T = 300 K along with the result of the Rietveld refinement of the tetragonal P4_2_/mnm crystal structure (**b**) bulk NiF_2_ crystal structure with tetragonal coordination of Ni (light yellow circled) with F (red circled) atoms for clarity.

Rietveld refinement is carried out using FullProf program^21,22^ and the refined pattern is shown in Fig. 1(a). The refined structure is tetragonal with P4_2_/mnm space group (No. 136) and all the respective Bragg diffraction positions are shown in Fig. 1(a). The lattice parameters are a = 4.68 Å = b and c = 3.06 Å and the respective crystal structure is shown in Fig. 1(b). The structure consists of NiF_6_ octahedra, sharing edges along the c - direction and corners in the a-b plane. The position of nickel and fluorine atoms are (0 0 0) and (0.30183 0.30183 0.00000) in the unit cell^20^.

The theoretically computed lattice parameters are a = 4.70 Å and c = 3.12 Å, in good agreement with refined structural parameters, and consistent with the reported literature^23,24^. The transition atom is coordinated with six fluorine atoms which form the distorted octahedral symmetry because of two different Ni atomic sites, leading to different Ni-F bond lengths (Fig. 1(b). The computed distorted octahedral is consistent with the structural refinement results and the structure is thermodynamically robust, as supported by the computed phonon dispersion, see Supplementary Information (SI).

Further, we considered three different magnetic ordering of Ni atoms in NiF_2_: (i) paramagnetic (ii) ferromagnetic and (iii) antiferromagnetic ordering, in order to understand the magnetic structure. The ground state energy is the lowest for antiferromagnetically ordered Ni sites in the NiF_2_ system. This is consistent with the experimental observations, where NiF_2_ is showing AFM transition from high-temperature PM phase at or below 68.5 K. The experimental observations suggest the onset of another metastable FM phase at or below 10–12 K. The corresponding electronic properties are computed to understand the contribution of different atomic orbitals constituting different magnetic structures (see SI).

**Figure 2:**
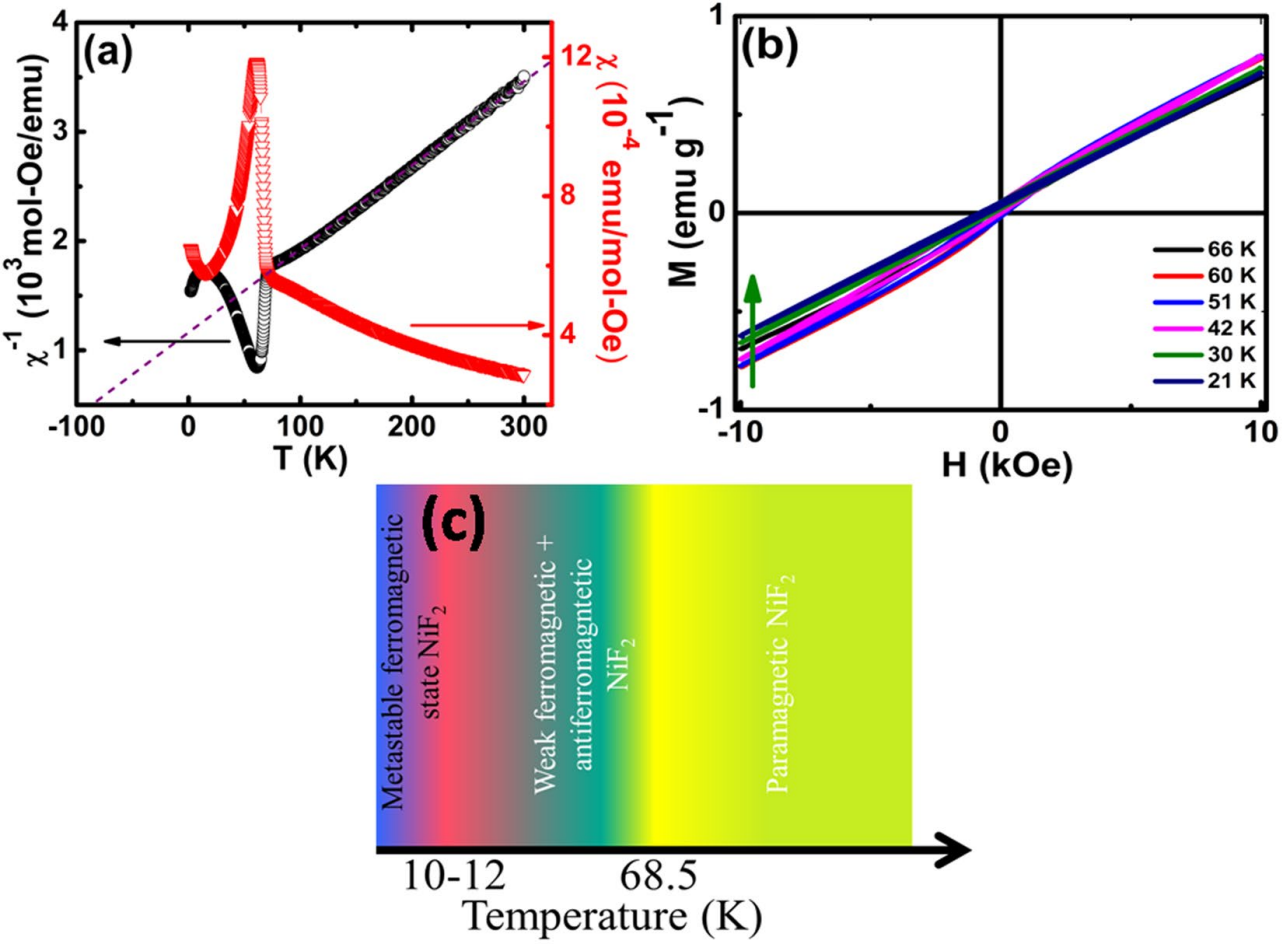
(**a**) Temperature dependent magnetic susceptibility (red curve) and inverse magnetic susceptibility (black curve) for NiF_2_ bulk sample. (**b**) Magnetization vs. field at different temperatures below AFM ordering temperature ~68.5 K. (**c**) Schematic magnetic phase diagram of NiF_2_ bulk.

The temperature dependent magnetic measurements indicate an AFM state near T_N_ = 68.5 K in Fig. 2(a). The sharp rise in magnetic susceptibility near this AFM transition signifies the presence of weak FM state as well^25^. This may be attributed to the canted Ni spin structure in NiF_6_ octahedra, as explained earlier in structural properties. The inverse magnetic susceptibility as a function of temperature is also plotted. Curie–Weiss law, χ = C/(T − Θ_CW_), where χ is magnetic susceptibility, C is the Curie-Weiss constant and Θ_CW_ is Curie-Weiss temperature, fit in the PM region from 100 K–300 K and the corresponding fit is shown using a dashed black line. The extracted Curie–Weiss temperature Θ_CW_ is ~−83.74 K, suggesting the long-range AFM ordering. The frustration index f (=|Θ_CW_/T_N_|) is near unity (~1.22), substantiating the absence of magnetic frustration and thus, ruling out the possibility of any magnetic clustering in the system. The observed sharp rise in magnetic susceptibility near AFM transition is not because of any localized magnetic impurity and is attributed to the spin canting at nickel sites. The estimated Curie-Weiss constant C is 7.71 emu K mol^−1^, equivalent to ~1.02 μ_B_ effective PM moment. The calculated PM moment is close to μ_eff_ = 1 μ_B_, for S = 1 Ni^2+^ ions^20^.

In addition to the observed AFM transition, superimposed with canted Ni site spin driven weak FM state, NiF_2_ is showing an additional magnetic ordering on reducing the temperature below ~10 K, where the magnetic moment showed enhancement with lowering temperature. This may be attributed to the spin reordering at the cost of the internal magnetic field against temperature, leading to another FM ordering state. Further, low-temperature magnetic hysteresis measurements are carried out to probe the magnetic nature of NiF_2_ in Fig. 2(b).

The observed nonlinear magnetization as a function of field substantiates the presence of weak FM ordering in conjunction with long-range AFM ordering of NiF_2_. This is consistent with the observed sharp rise in magnetization near AFM transition at T_N_. The magnetic moment at 10 kOe field near T_N_ is the maximum as compared to that of at lower temperatures. The decrease in the magnetic moment against temperature is attributed to the robustness of AFM ordering at lower temperatures, whereas near T_N_, the dominant spin canting at Ni sites is giving rise to the observed FM contribution^26^.

Further, the computed total energy values are summarized in Table 1 for different magnetic structures. The energy of AFM NiF_2_ is the lowest among all investigated structures, substantiating that the ground state of NiF_2_ is AFM. The total energy of PM NiF_2_ is the highest and is in agreement as system shows the transition from PM state to AFM state at 68.5 K as the calculations are carried out at 0 K. The more interesting part is that the total energy of FM state is close to AFM state and the total energy difference is very small ~−0.043845 eV. The comparable energy of FM state suggests that this state may also exist simultaneously with AFM state and is substantiating the experimental observations, where NiF_2_ is showing another FM transition near ~10 K. Further, the total magnetic moment of unit cell is about 4 μ_B_, with partial magnetic moments 1.92 μ_B_ and 0.05 μ_B_ contributed by Ni and F atoms, respectively. The results are consistent with reported by Das *et al*.^18^.

**Table 1:** Total energy, magnetic moment and band gap values for NiF_2_ in different magnetically ordered structures (NM, FM and AFM stand for nonmagnetic, ferromagnetic and anti-ferromagnetic structures).

Properties		NM	FM	AFM
Total energy (eV)		−6864.54906272	−6864.92857441	−6864.97241960
Magnetic moment	Total	0 μ_B_	4 μ_B_	0 μ_B_
	Partial	Ni = 0 μ_B_, F = 0 μ_B_	Ni = 1.92 μ_B_, F = 0.05 μ_B_	Ni_1_ = −1.91 μ_B_, Ni_2_ = 1.91 μ_B_ F_1_ = −0.02 μ_B_, F_2_ = 0.02 μ_B_
Band gap	Spin up	6 eV	9.23 eV	8.07 eV
	Spin down	6 eV	7.80	8.07 eV

Additionally, the total magnetic moment is zero in AFM state with partial magnetic moments Ni_1_ = −1.91 μ_B_ and Ni_2_ = 1.91 μ_B_ with nearly zero (0.02 μ_B_) magnetic moment on fluorine atoms. The respective magnetic moments are summarized in Table 1. The complex magnetic behavior of bulk NiF_2_ is summarized schematically in Fig. 2(c), showing the different magnetic phases against temperature.

**Figure 3:**
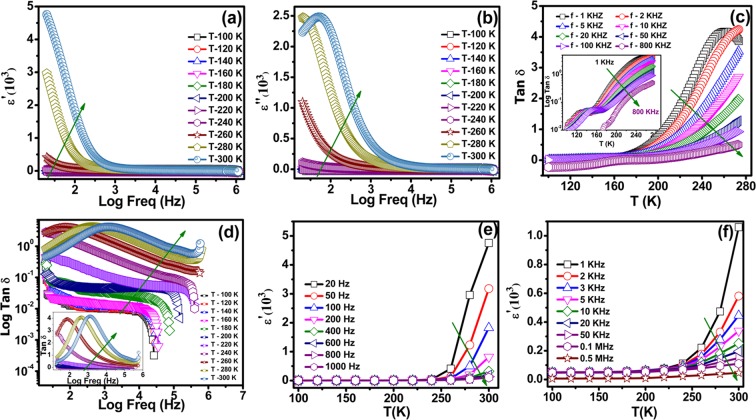
(**a**) The real part of the dielectric constant versus frequency. (**b**) The imaginary part of the dielectric constant versus frequency. (**c**,**d**) The loss component of the dielectric response, expressed as Tan δ. (**e**,**f**) Temperature dependent dielectric constant is measured at low and higher frequency range from 100–300 K.

We probed the temperature and the frequency dependent dielectric properties. The dielectric dispersion, as shown in Fig. 3(a), suggests that real dielectric constant changes drastically with temperature and frequency. The dielectric constant ‘ε’ values, Fig. 3(a,b), tend towards a very large number with increasing temperature at lower frequencies. It is interesting to note in Fig. 3(c,d) the very small values of the loss tangent over the entire temperature and frequency range, suggesting the intrinsic feature of the material. Further, we notice two temperature ranges, where dielectric relaxation is observed. The loss tangent values, Tan δ = ε”/ε’, where δ is the phase difference between applied electric field and induced current, is showing the first peak near 150 K and a second peak near 280 K, as can be seen in Fig. 3(c,d)^27^. The onset of this dielectric loss is attributed to the lag in dipole polarization behind the applied alternating field, which may be caused by grain boundaries, impurities and imperfections in the crystal lattice^28^. These observations substantiate the dielectric relaxation in NiF_2_ bulk sample and are discussed later. Further, the dielectric constant values, Fig. 3(a,b), reduced to much lower nearly constant values with increase in frequency. The high frequency values are temperature insensitive^29^. Also, the observed rapid fall in dielectric constant (ε′) near 1 kHz may suggest a fractional contribution of the space charge polarization to the total polarization observed in the NiF_2_ system. The space charge polarization decreases in ionic and orientation polarizability with increasing frequency, which may be responsible for the observed decreases in ε’ at lower frequencies, in agreement with the reported literature^30^. Phase charge polarization, also called interfacial polarization, arises whenever different conductivity phases are present in the same material. When an electric field is applied, the charges move through a conducting phase but are interrupted as they come across a high resistivity phase^31^. Temperature-dependent dielectric constant in low and high frequency region Fig. 3(e,f) shows, ε’ increases with increasing temperature and decreases with increasing frequency. Further, the value of ε’ becomes less below 220 K and it becomes temperature independent down to100 K. It means that dipoles were frozen (relax out) at low temperature regions. Commonly, the capacitive effect arising due to the semiconducting nature of grain and the grain boundaries is considered as one of the possible source of high dielectric constant and originating from the presence of permanent dipole moments.

**Figure 4:**
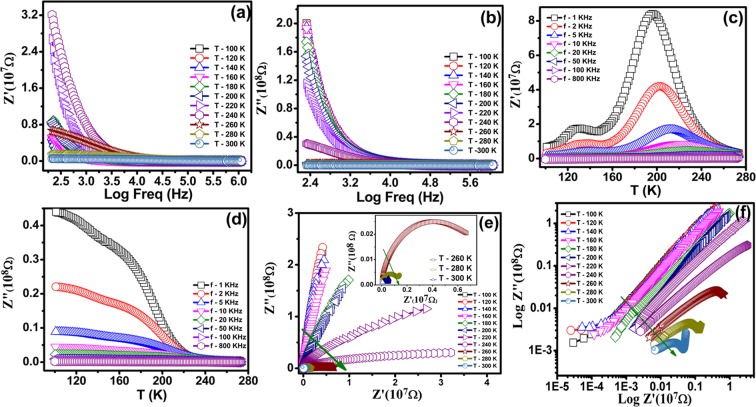
Impedance spectra of NiF_2_. (**a**) Resistive (Real). (**b**) Reactive (Imaginary) Impedance Spectra. (**c**,**d**) Impedance of real and imaginary at different temperatures. (**e**,**f**) Complex impedance spectra of NiF_2_ at different temperature.

Further, temperature and frequency dependent impedances are analyzed for NiF_2_ bulk sample. The semi-log frequency versus real and imaginary impedance components are plotted in Fig. 4(a,b), respectively for various temperatures. The impedance values (both real and imaginary components) are too large in the low-frequency range up to ~1 kHz, which further converge to low values for all temperatures with increasing frequency.

The signature of any transition in these data is difficult to visualize and that’s why impedance (both real and imaginary components) is plotted against temperature at various frequencies in Fig. 4(c,d). These measurements clearly suggest the two impedance peaks, near 170 K and 270 K, substantiating the similar observations from loss tangent versus temperature measurements, (see Fig. 3c,d), as discussed earlier. The observations of these peaks suggest the presence of two dielectric relaxation windows. Further, peak temperatures (for both peaks) are shifting towards higher values with increasing frequency, and simultaneously the intensity of peak is decreasing with increasing temperature. The observed reduction in peak intensity and increase in frequency with temperature support that both dielectric relaxation responses are thermally activated. The representative semicircular impedance nature is associated with conduction in linear complex impedance plots and is distorted in the present case, Fig. 4(e), for all temperatures, down from 100 K till 300 K. However, a signature of the semicircular impedance curve can be observed in complex impedance plots, Fig. 4(f) ^32^.

**Figure 5:**
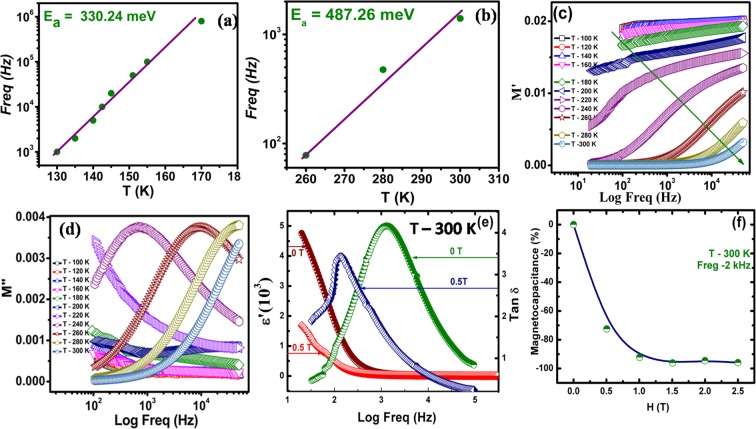
(**a**,**b**) Activation energy of NiF_2_ in the temperature range of 130 K–170 K and 260 K–300 K. (**c**,**d**) Frequency dependence of (**c**) Real (*M*′ versus Freq) (**d**) imaginary parts (*M*″ versus Freq) of electric modulus at different temperatures. (**e**) Dielectric constant and loss versus frequency with respective applied magnetic field and (**f**) calculated magneto capacitance for the NiF_2_ material at room temperature.

The radius of the semicircle is small for higher temperatures i.e. the least for 300 K impedance measurement (~4.6 × 10^5^ Ω), which increases further with reducing temperature, as can be noticed in Fig. 5(b). Further, the radius of even first semicircle gets too large below 260 K and is measurable up to 260 K only. The radius of the first semicircle is reducing continuously with increasing temperature, with a shift towardsthe left from the right side, which is attributed to the observed reduction in impedance with temperature. The observations are similar to that of several materials such as Bi_2/3_Cu_3_Ti_4_O_12_^33^ and CaCu_3_Ti_4_O_12_^34^.

The impedance of powder samples is analyzed using resistance ‘R’ and capacitance ‘C’ based equivalent circuits. The data is usually modeled as a series combination of two parallel networks, one consisting grain resistance ‘R_g_’ and grain capacitance ‘C_g_’, while other consists of grain boundary resistance ‘R_gb_’ and grain boundary capacitance ‘C_gb_’. Each parallel network results into a semicircular arc, with lower impedance arc represents the grain contribution, whereas higher impedance arc represents the grain boundary contribution^33,35^. Most surprisingly, in our studies, we could observe only the first lower impedance semicircular arc, whereas the second larger impedance semicircular arc may correspond to the range beyond the measurements. Thus, we avoided simulating the impedance data using the grain and grain boundaries model. In addition, the distorted nature of relatively low impedance data hampers simulating the effective contributions from grain and grain boundaries. We extracted the temperature dependence of peak frequency for both temperature dependent relaxation processes and are summarized in Fig. 5(a,b). The semi-logarithmic plots are used for frequency versus temperature data to understand the relaxation process^36^. These relaxation processes follow Arrhenius behavior as *f* = *f*_0_ *exp* (−*E*_*a*_/*k*_*B*_*T*), where *f*_0_ is prefactor and *E*_*a*_ is the activation energy for thermal response and *k*_*B*_ is Boltzmann constant and T is the temperature in K. The straight line fit is shown in respective plots, Fig. 5(a,b). The activation energy values are 330.24 meV and 487.26 meV for low and high temperature relaxation processes, respectively.

The observed impedance peaks are relatively poor in complex impedance spectroscopy, attributed to the relatively smaller resistance grains with respect to that of grain boundaries in general for mixed impedance contribution from both grain and grain boundaries^37^. Considering the same, the electrical modulus *M* = *M*′ + *iM*″ (=*iωC*_0_*Z*, where *C*_0_ (=*ε*_*0*_*A/d*) is the equivalent sample cell capacitance in vacuum, *A* is the electrode area and *d* is the sample thickness, *Z* is impedance, *ω* is angular frequency (=2*πf*), *M*′ and *M*″ are real and imaginary component of electrical modulus) is used to separate out the grain and grain boundary contributions in the effective dielectric properties^38^. This approach is useful in understanding the microscopic origin of conduction/relaxation processes, activated in the system. The computed real and imaginary components of electrical modulus are plotted in Fig. 5(c,d). The frequency dispersion of the electrical modulus is large and both real as well as imaginary component exhibit strong frequency dependence. The frequency dependence is used to distinguish the contribution of grains and grain boundaries in effective dielectric values. Further, the observation of broad peaks in *M*″ and its variation is attributed to the non-uniform micro-structured grains in bulk sample, causing variations in local electronic conductivities and to the diffusive ionic motion in these grains. The variation of peak frequency with temperature provides the clear evidence for these effects. If peak frequency is decreasing with increasing temperature, grain boundary effect is contributing to the effective dielectric constant, whereas the reverse substantiates the grain effect contribution to the effective dielectric constant. More interestingly, we observed that peak frequency is increasing with increasing temperature, Fig. 5(d), suggesting the grain effect is dominating in the measured dielectric response for the investigated temperatures (up to 300 K in present studies). The clear peaks are observed for temperature 240 K and above, where peak frequency is increasing with increasing temperature. The peak of the maxima corresponds to 2*π f*_*max*_ *τ* = *1* in *M*″ versus frequency measurements, where *τ* is the response time and *f*_*max*_ is the peak frequency. The response time is about 10 μ*s* at 240 K temperature, which is substantially large which may be the main reason for not observing the clear peaks for low-temperature measurements. The peak frequency is reducing with temperature, suggesting that response time will increase from microseconds to milliseconds or seconds and causing the peak frequency to shift towards much lower frequency and that’s why the lower temperature peak frequencies are not observed in the present measurements. The activation energy estimated from the response time versus temperature measurements is ~620.63 meV, which is slightly higher than that of 487.26 meV, estimated from the variation of the tangent loss versus frequency measurements, Fig. 3(d). The reason for the observed difference is attributed to the broader frequency peaks, posing difficulties in estimating the clear peak frequency. In spite of the different values, the large activation energy substantiates that the observed relaxation is intrinsic and grains are mainly contributing to the observed dielectric response in the NiF_2_ bulk sample. Thus, these studies may provide a new materials system, where intrinsic grain effect is responsible for the observed large dielectric response. Also, we find that the dielectric permittivity strongly depends on the applied magnetic field. In order to understand the origin of the charge and phase separation behavior, we carried out the studies of the effect of magnetic field on the frequency dependent dielectric properties has shown in Fig. 5(e,f). The frequency dependence of ‘*ε*’ in the NiF_2_ at the external magnetic field 0 T and 0.5 T at the field cooling regime (FC) shown in Fig. 5(e). The magnetic dielectric constant and dielectric loss marginally decreased with respect to the applied magnetic field of 0.5 T at room temperature. In this condition, the magnetic field stimulates the occurrence of the homogeneous magnetic order. The magnetic field dependent magnetocapacitance is shown in Fig. 5(f) up to 2.5 T magnetic fields. The magnetocapacitance behavior could be understood by the effect of magnetic field on the properties of the charge separation structure. The magnetic field decreases the magnetic ordering and the charge ordering^39,40^ and leads to change the magnetocapacitance^41^ $MC\,( \% )=\frac{\varepsilon ^{\prime} (B)-\varepsilon ^{\prime} (0)}{\varepsilon ^{\prime} (0)}\times 100$. The increasing magnetic field led to the enhanced negative magnetocapacitance >90% is noticed in a field of 1 T at (2 kHz) at room temperature has shown in Fig. 5(f).

In summary, we have investigated the magnetic structure and dielectric response of the bulk tetragonal (P4_2_/mnm space group) compound NiF_2_, showing an AFM transition near T_N_ = 68.5 K from high-temperature PM phase. This is followed by a weak signature of low-temperature FM transition near 10 K. A representative magnetic phase diagram against temperature is predicted and the low-temperature phase is termed as metastable FM state. Further, the canted spin of Ni atoms exhibit a weak FM signature superimposed on AFM state. More interestingly, a colossal dielectric response is observed with dielectric constant ~10^3^ at about 20 Hz near room temperature. The frequency dependence measurement substantiates the intrinsic grain effect for observed such response. These studies demonstrate that the non-oxide NiF_2_ is a promising multifunctional material, showing rich magnetic, dielectric and negative magnetocapacitance (>90%) properties. The observation is large magnetocapacitance is attributed to the non-homogeneous grain distributions in NiF_2_ bulk materials^42^. We hope our data would incite others to look for such effects in other non-oxide systems.

## Methods and Characterization

Commercial powder of NiF_2_ (97% anhydrous) was obtained from Alfa Aesar. Phase identification of NiF_2_ was carried out by powder XRD using a Smartlab, Rigaku X-ray diffractometer. The X-ray generator was operated at 40 kV and 30 mA. Diffraction data were collected by step scanning method over an angular range of 20–80° with a step range of 0.01°. The magnetization measurements were carried out using a PPMS-VSM (Model: 6000, Quantum Design, USA). For the dielectric measurements, the powder was pressed in a pelletizer under a pressure of 6 Ton using a 20 Ton hydraulic press. The resultant pellet has a thickness of 1.25 mm and 13 mm in diameter. For dielectric measurements, the conducting layer of silver was deposited on both the sides of the pellets to have better ohmic contacts. A programmable Precision LCR bridge (Key sight: E4980A) has been used to measure the dielectric properties in the frequency range from 20 Hz to 1 MHz from 300 K to 100 K. The density functional calculations are carried out for detailed structural, optical, electronic, and magnetic properties using anaugmented plane wave plus local orbitalsmethod, as implemented in Wien2K. The details are provided in supplementary information^43^.

## Supplementary information

Complex magnetic structure and magnetocapacitance response in a non-oxide NiF2 system
