# Supplementary material for: Complex magnetic structure and magnetocapacitance response in a non-oxide NiF2 system
Source: Sci Rep. 2019 Mar 1;9:3200. doi: 10.1038/s41598-019-39083-8 (PMC6397207; doi:10.1038/s41598-019-39083-8)
Supplement: Supplementary file 1 — Complex magnetic structure and magnetocapacitance response in a non-oxide NiF2 system [file 41598_2019_39083_MOESM1_ESM.docx]

Supplementary Information

Complex magnetic structure and magnetocapacitance response in a non-oxide NiF_2_ system

S. Arumugam^a,*^ P. Sivaprakash^a^, AmbeshDixit^b^, Rajneesh Chaurasiya^b^, L. Govindaraj^a^,

M. Sathiskumar^a^, SouvikChatterjee^c^, R. Suryanarayanan^d,e^

^a^Center for High Pressure Research, School of Physics, Bharathidasan University,

Tiruchirappalli, 620 024, India.

^b^Department of Physics & C for Solar Energy, Indian Institute of Technology Jodhpur, 342 037, India.

^c^UGC-DAE Consortium for Scientific Research, Kolkata Centre, Kolkata 700 098, India.

^d^(Retired) ICMMO, University of Paris- Sud, Orsay, 91405 France.

^e^ Present address 3, allée des Marronniers, Les Ulis, 91940 France

^*^E-mail: [sarumugam1963@yahoo.com](mailto:sarumugam1963@yahoo.com)

**Computational details:**

The density functional calculations are carried out under full potential linear augmented plane wave (FP-LAPW), as implemented in Wien2K**^1^**.The modified Becke-Johnson parameterization is used as an exchange correlation function **^2^**. The unit cell is divided in muffin tin region (with R_mt_ as radius) and interstitial region (IR). The muffin tin radii for nickel and fluorine atoms are chosen in such a way that there is no overlap among different atomic elements. The plane wave cut off parameters R_mt_*K_max_= 7 and G_max_ = 12 are used for structural, electronic and magnetic properties of NiF_2_. The maximum value of *l* (*l*_max_) is considered 10 and cut-off energy is at − 6.5 Ry, defining the separation between the core and valence states. The self-consistent calculations are carried out under total energy convergence of∼ 0.001 Ry. A large plane wave cut-off of 150 Ry is used throughout the calculation and initially 125 k-points are considered in Brillouin zone for computing the structural parameter while 1000 K point are used for computing the other properties like electronic and magnetic properties of the material.

**Phonon band dispersion and electronic/optical properties:**

We further computed phonon structure for optimized NiF_2_ bulk system to probe the thermodynamic stability and plotted in Fig 1. The absence of imaginary frequencies substantiate that the structure is thermodynamically robust. The eighteen phonon modes are observed at Γ, as the unit cell consists of two NiF_2_ formula units. The lowest energy three modes at Γ are acoustic modes (K🡪 0, ω🡪 0) and the rest are optical modes (K🡪 0, ω ≠ 0).

The calculated band structure of paramagnetic NiF_2_ is shown in Fig.2 with partial density of states and room temperature Tauc plot for bulk NiF_2_ powder sample. The Fermi energy is set at 0 eV, Fig.2 (left panel). We observed the direct band gap i.e. valence band maxima and conduction band minima are located at Γ in Brillouin zone, with band gap value ~ 6 eV, Fig.2 (left panel), consistent with experimental measurements, as shown in Tauc plot, Fig.2 (right panel). The computed partial and total density of states are shown in Fig.2 (middle panel), suggesting that valence band is made of Ni d_xz_, Ni d_x_2_-y_2, Ni d_z_2 and F p states, while conduction band consists of Ni s states. More interestingly, we also observed a strong absorption peak near 3 eV in conjunction with large band gap, Fig 2 (right panel). This strong absorption is associated to Ni intra band transitions as can be observed from partial density of states. We observed that the presence of Ni d_x_2_-y_2, Ni d_z_2 orbital’s within the band gap of NiF_2_ near 3 eV, substantiating the observed strong absorption peak in Tauc plot.

Further, we carried out electronic band structure and density of states (both partial and total) for both ferromagnetic and anti-ferromagnetic Ni spin ordering in NiF_2_. The band structure and density of states are shown in Fig.3 for ferromagnetic NiF_2_, showing electronic band structure for spin up (black color) and spin down (red color) states. The band gap values are 9.23 eV and 7.80 eV for spin up and spin down states, respectively, Fig 3 (left panel).

The change in electronic band gap from the paramagnetic state is observed due to the onset of exchange splitting between Ni-d and F-p orbitals. The partial densities of states are shown in Fig.3 (middle and right panel) for both spin up and down states in conjunction with total density of states. The valence band for spin up states consists of Ni-d orbitals near Fermi energy and F-p orbitals around 6–9 eV far from Fermi energy, Fig 3 (middle panel). The nickel Ni-d_z_^2^, Ni-d_x_^2^_y_^2^, Ni-d_xy_ and Ni-d_yz_ orbitals constitute valence band for spin up states, Fig 3 (right panel) with Ni-d_z_^2^, Ni-d_x_^2^_y_^2^, and Ni-d_xz_ orbital’s forming conduction band minima, Fig. 3 (right panel).

The anti-ferromangenetic spin polarized electronic band structure is shown in Fig.4 (left panel), showing similar electronic band structure for both spin up and spin down states. The band gap value is 8.07eV, which is relatively larger than that of paramagnetic NiF_2_ and smaller than that in ferromagnetic state, as discussed above. The partial densities of states are shown in Fig. 4 (middle panel) and Fig.6 (right panel) for spin up and spin down states, respectively. The valence band of antiferromagnetic NiF_2_ consists of Ni_2_-d orbital in conjunction with small contribution from Ni_1_-d orbitals, whereas conduction band consists of Ni_2_-dz^2^, Ni_2_-d_x_^2^_y_^2^ and Ni_2_-d_yz_ orbitals. Moreover, the spin down DOSs is shown in Fig. 4 (right panel), confirming that valence band near the maxima is mostly dominated by Ni_1_-dz^2^, Ni_1_-d_x_^2^_y_^2^, Ni_1_-d_xy_ and Ni_1_-d_yz_ orbitals and conduction band minima are due to Ni_1_-dz^2^, Ni_1_-d_x_^2^_y_^2^ and Ni_1_-d_xz_ orbitals. The Ni s-states are contributing towards deeper in the valence band.

The capacitance and tangent loss data are recorded as a function of frequency for different temperatures on a thick NiF_2_ pellet. The measured capacitance is converted into real dielectric constant ε_r_ = C.d / ε_0_.A, where C is the measured capacitance in Farad (F), d is the thickness in meter, ε_0_ is free space permittivity (= 8.854 x 10^-12^ F.m^-1^) at different frequencies and A is the electrode area. The results are summarized (see main text Fig. (3)) for 20 Hz-1 MHz frequency range at different temperatures ranging from 100 K to 300 K.

**Reference:**

1. Peter Blaha, Karlheinz Schwarz, Georg K. H. Madsen, Dieter Kvasnicka, Joachim Luitz:WIEN2k, *revised edition WIEN2k 08.3 (Release 18/9/2008)*, ISBN 3-9501031-1-2.
2. David Koller, Fabien Tran, and Peter Blaha. Improving the modified Becke-Johnson exchange potential. ***Phys. Rev. B 85, 155109 (2012).***

**Figure Captions:**

**Figure 1:** The phonon dispersion for bulk NiF_2_ system, showing dynamic stability and 18 phonon bands at Γ point.

**Figure 2:** Band structure (left panel), partial density of states (mid panel) for nonmagnetic NiF_2_ bulk and room temperature Tauc plot ((α.E)^2^ versus energy) for paramagnetic NiF_2_ bulk powder sample (right panel) with inset showing reflectance versus wavelength.

**Figure 3:** Electronic band structure (left panel); spin up (middle panel) and spin down (right panel) partial density of states for NiF_2_in ferromagnetic (FM) state.

**Figure 4:** Electronic band structure (left panel); spin up (middle panel), and spin down (right panel) partial density of states for NiF_2_in antiferromagnetic state.

**Figure 1**


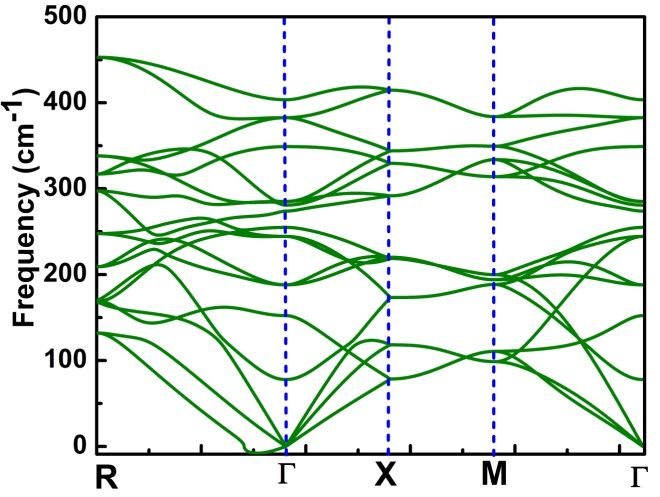


**Figure 2**

**
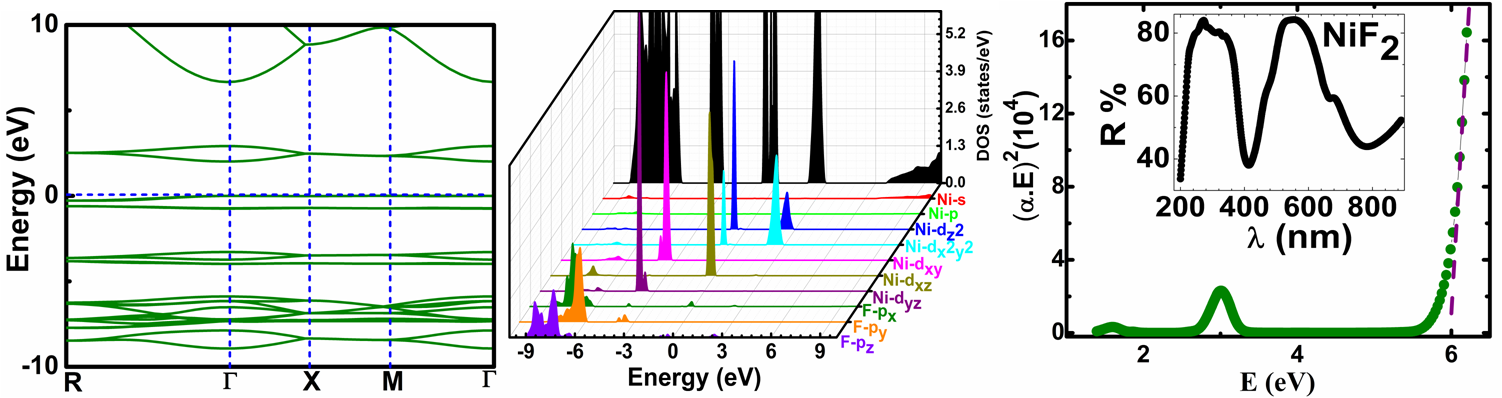
**

**Figure 3**

**
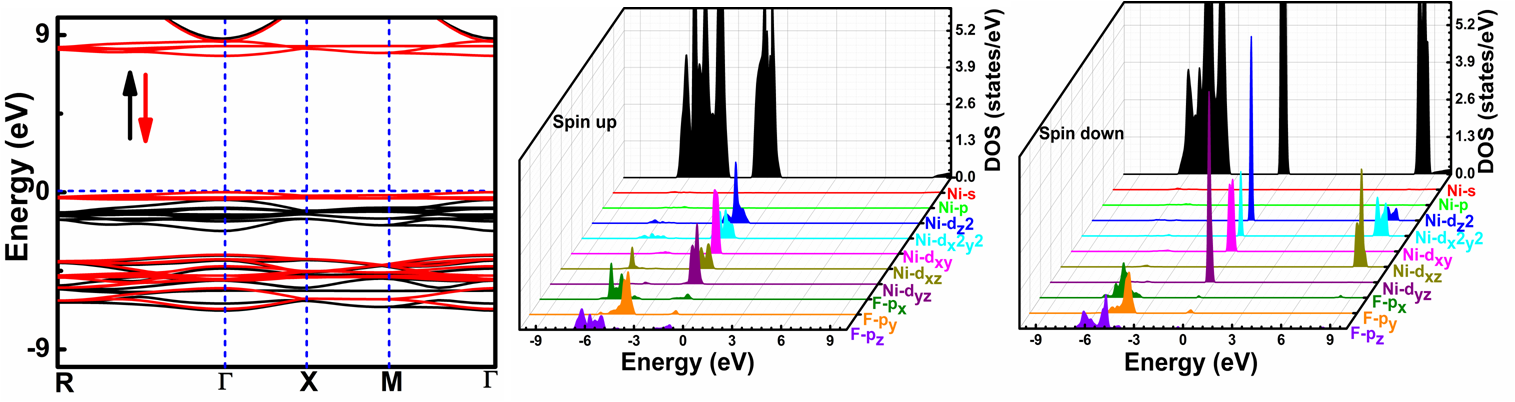
**

**Figure 4**

**
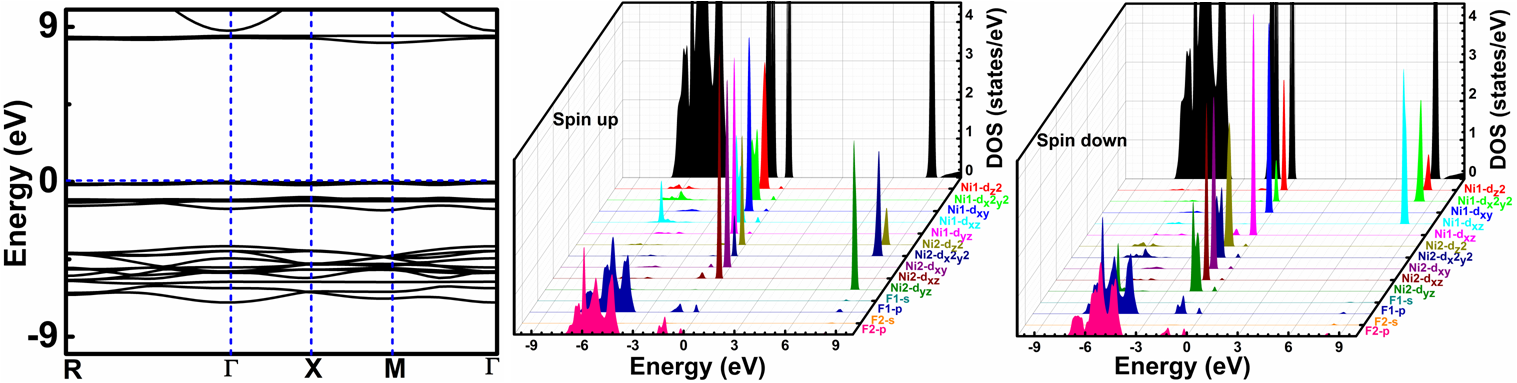
**
